# A Review of Fetal Scarless Healing

**DOI:** 10.5402/2012/698034

**Published:** 2012-05-17

**Authors:** K. J. Rolfe, A. O. Grobbelaar

**Affiliations:** Institute for Plastic Surgery Research and Education, The Royal Free Hospital, Pond Street, Hampstead, London NW3 2QG, UK

## Abstract

Wound healing is a complex process involving a number of processes. Fetal regeneration has been shown to have a number of differences compared to scar-forming healing. This review discusses the number of differences identified in fetal regeneration. Understanding these differences may result in new therapeutic targets which may reduce or even prevent scarring in adult healing.

## 1. Introduction

Since the 1970s it has been well established that early human fetuses can heal cutaneous wounds without the formation of scar tissue [1]. This regeneration appears organ specific, as in early fetuses which heal cutaneous wounds with perfect regeneration other organs such as the gut heal with the formation of scar tissue. Studies on the marsupial embryo, *Monodelphis domestica*, have shown that fetal regeneration is not due to the moist, sterile environment of the uterus [2]. Further, this regenerative phenotype is cell specific with fetal skin transplanted subcutaneously onto adults continuing to show a regenerative phenotype [3] whereas skin from the adult transplanted onto the fetus demonstrate an adult-like scarring phenotype [4].

Wound healing is an inherent response resulting in restoration of tissue integrity. It is a complex process involving cell migration, proliferation, differentiation, apoptosis, and the synthesis and remodelling of the extra cellular matrix (ECM). A number of factors are involved in the various stages of tissue repair including cell-cell interactions, cell-matrix interactions, a number of different cell types, and a large number of growth factors and cytokines. The regenerative phenotype of the fetus has shown a difference in a number of processes involved in wound healing, which may be manipulated to reduce or even prevent scarring.

## 2. Inflammation

Fetal wound healing compared to adult wound healing has been shown to have a different and reduced inflammatory response [5, 6]. The levels of immune cells are reduced which include macrophages, which are also less activated, and, in addition, the presence of inflammatory cells is short lived in fetal wound healing compared to the adult [5–7]. The reduced number of inflammatory cells also means lower expression levels of some growth factors and cytokines and for a shorter duration of time [8, 9]. However, studies have shown that fetuses which are artificially stimulated to produce an inflammatory response show an adult-like response with scar formation [10, 11]. It appears that no single immune cell is essential for wound healing [12–14] with PU.1 knockout mice, which lack both macrophages and neutrophils, showing improved rates of reepithelisation and reduced scarring compared to their wild-type equivalents [15].

The proinflammatory cytokines interleukin-6 (IL-6) and interleukin-8 (IL-8) have been found to be decreased during scarless fetal repair even when fetal fibroblasts are stimulated with platelet-derived growth factor (PDGF) [16, 17]. IL-10 is known to be a major regulator in suppressing the inflammatory response, including IL-6 and IL-8, and IL-10 also inhibits the migration of inflammatory cells to sites of injury [18–22]. Knockout animals for IL-10 demonstrate scar formation in fetal wounds which would have healed without a scar [23] while over expression of IL-10 in adult wounds, using genetic manipulation, decreased the inflammatory response, decreased abnormal collagen deposition, and restored normal architecture [24].

Cyclooxygenase-2 (COX-2), part of the arachidonic acid cascade, is upregulated in response to an inflammatory response such as an injury. COX-2 functions by producing prostaglandins which control many aspects of inflammation. A murine model of scarless healing demonstrated low levels of COX-2 and prostaglandin-2 (PGE2), whereas the addition of exogenous PGE2 induced scar formation in a fetal model of wound healing [25]. Blocking the COX-2 enzyme in adult wounds results in a fetal-like phenotype with reduced scarring [26]. However, both fetal and adult fibroblasts show expression of the PGE2 receptors [27]. PGE2 inhibited fibroblast migration, in both the fetus and adult, through the EP2/EP4-cAMP protein kinase A pathway, though fetal fibroblasts appeared refractory requiring a higher concentration to achieve the same effect. The inhibition of adult fibroblast migration by PGE2 correlated with the disruption of the actin cytoskeleton, and PGE2 also inhibited the contraction of adult derived fibroblast populated collagen lattices. PGE2 however, did not disrupt the actin cytoskeleton in fetal-derived fibroblasts and further did not prevent fetal fibroblast populated collagen lattices contraction [27], possibly because fetal fibroblasts are thought to have a more migratory phenotype [28].

## 3. Extra Cellular Matrix (ECM)

The ECM is known to play an important role in wound healing as it can play a part in regulating growth factors and cytokines and alter cell behaviour [29]. Fetal wounds have been shown to have increased levels of glycosaminoglycans such as hyaluronic acid (HA) and chondroitin sulfate, which are long unbranched polysaccharides comprising of repeating disaccharides found on the cell surface or in the ECM. HA is found at higher levels and for a longer duration in fetal wounds compared to adult wounds [30, 31]. This increased expression is possibly due to the reduced activity of hyaluronidase in the fetus [32] while fetal fibroblasts also express higher levels of the hyaluronic acid receptor (CD44) compared to adult fibroblasts [33]. Exogenous addition of HA reduces the formation of scar tissue in adults [34, 35] while reducing HA expression results in a phenotype more akin to adult healing [36]. Increased levels of HA as identified in the fetus promotes both the proliferation and migration of a number of cell types [37]; HA-rich matrices can bind growth factors and cytokines which can result in temporal and spatial differences of these factors.

Glycoproteins, such as fibronectin, laminin, and tenascin C, bind integrins, collagen, and proteoglycans and are integral components of the ECM playing a role in cell adherence [38]. Fibronectin is involved in the migration of a number of cells involved in wound healing including fibroblasts, keratinocytes, and endothelial cells. The fibronectin family consists of numerous splice variants in humans with a number of variants being involved in both fetal development and wound healing [39–41]. Fibronectin which is part of the provisional matrix, shows similar temporal and spatial expression in both fetal and adult sheep and mice [42, 43] while another animal model (rabbit) suggests that fetal wounds show an earlier expression of fibronectin [44]. Tenascin C has shown earlier deposition in fetal wounds which may be associated with the rapid reepithelisation seen in fetal wounds [42, 43]. The wounded fetal human skin has shown increased expression of integrin subunits *α*2, *α*3, *α*6, and *β*4, (laminin and collagen receptors) and neoexpression of *α*5, *α*V, and *β*6 (fibronectin and tenascin C receptors), and this may further explain the fetuses' ability to reepithelise wounds rapidly with a reduced presence of inflammatory cells [45].

The proteoglycans decorin and fibromodulin which are known to regulate collagen fibrillogenesis, growth factor activity, and cellular proliferation have shown variation in fetal wound healing. Decorin showed reduced expression in fetal fibroblasts and fetal skin compared to adult fibroblasts and skin [46]. While decorin was upregulated during adult wound healing, it has also been shown that reduced or delayed expression of decorin is associated with pathological scarring in a number of adult models [47, 48]. Fibromodulin, a further proteoglycan, showed an increase protein expression in scarless wounds compared to scarring [49] and similarly to decorin [50] is believed to alter the biological activity of TGF-*β*.

Fetal and adult wounds show a number of differences in collagen synthesis; these differences include speed of deposition, variations in collagen ratios and quantity of collagen itself [51–53]. Studies suggest that fetal fibroblasts not only show increased collagen III expression, but the new collagen is deposited in a fine reticular or basket weave pattern similar to uninjured skin [54, 55]. However uninjured fetal skin does show increased collagen III compared to collagen type I [51–53]. Others have suggested that the collagen deposited by fetuses is less mature with less cross-linking reducing rigidity but not affecting tensile strength [52]. This reduced collagen cross-linking may be due to a lower expression of lysyl oxidase, which is known to play a role in both collagen cross-linking and influences collagen architecture [56]. Chin et al. [57] also showed that fetal fibroblasts show increased expression of the collagen receptor DDR1 thought to be important for both collagen expression and organization. Though fetuses may show increased collagen production they do not exhibit excessive collagen deposition, and this may be through rapid turnover of the ECM components.

 Fetal wounds show increased levels of the urokinase plasminogen activator and matrix metalloproteinases (MMPs) while their inhibitors (PAI-1 and TIMPs) are reduced during fetal wound healing [58–60]. Higher levels of MMPs result in matrix degradation compared to matrix deposition. Dang et al. [60] showed that scarless fetal healing expresses MMP-1, MMP-9, and MMP-14 mRNA quicker and at higher levels than fibrotic fetal wounds. While MMP-2 and TIMP1 and TIMP 3 expression are not altered during scarless healing, whereas fibrotic wounds show decreased levels of MMP2 but with an increase in TIMPs [60]. 

## 4. Myofibroblasts and Contraction

Fetal studies have indicated that, unlike adult wound closure, fetal wounds close through an actin cable which acts like a purse string [61]. This cable assembles within minutes of an injury and requires a GTPase, Rho, to reepithelise fetal wounds [62]. Studies have shown that this cable may contain myosin which acts in a zipper-like manner to close incisional wounds in fetal skin [62], and paxillin mRNA expression was upregulated and colocalised with actin in the fetus but not in the adult [63]. Adult wound closure involves active movements of connective tissue and epidermis. The adult wound contracts to bring the two sides of the wound edges in close proximity to allow the epidermis to migrate and cover the exposed connective tissue [64].

Granulation tissue is thought to play a considerable part in wound contraction in adult wound healing. Migrating adult fibroblasts are capable of generating some tensile strength to start contraction, and the myofibroblast (differentiated fibroblast expressing alpha smooth muscle actin) is the main cell responsible for wound contraction. Differentiation of fibroblasts to myofibroblasts requires a combination of growth factors, mechanical cues, and the presence of the EDA variant of fibronectin. The presence of myofibroblasts in fetal wounds remains controversial with a murine model showing no alpha smooth muscle actin expression (except associated with blood vessels [65]), which was further replicated in a fetal sheep model [66]. However, Cass et al. [67] did detect myofibroblasts in fetal wound healing but at earlier time points than in postnatal (scarring) wound healing. Furthermore, others in an *in vitro* study have shown that human fetal fibroblasts can differentiate into myofibroblasts when stimulated with exogenous TGF-*β*1 but again at earlier time points than postnatal fibroblasts [68].

## 5. Growth Factors

Growth factors and their receptors play a vital role in wound healing with a number of aberrations associated with abnormal wound healing such as pathological scarring. A number of growth factors have shown different expression in fetal or scarless wound healing compared to adult or scarring wound healing (Table 1).

The TGF-*β* family is multifunctional and is believed to be important in both tissue repair and scarring. The three isoforms of TGF-*β* are synthesized as latent precursors which require activation before they can exert their biological activity through binding to their heteromeric receptor complexes. Fetal wound healing has shown a rapid induction of TGF-*β*1 mRNA in fetal repair but at lower levels and with a more rapid clearance from the wound site compared to adult wounds [8, 69, 70]. Interestingly TGF-*β*2 levels, also considered to be profibrotic, was found to be lower in adult-like repair compared to fetal repair [69]. The third isoform, TGF-*β*3, is expressed in adult animal wound healing [69, 71], though its expression is delayed [69], and with lower levels [69] compared to fetal wounds and in *in vitro* studies [68]. Studies have shown that blocking TGF-*β*1 and TGF-*β*2 may reduce scar tissue formation [72, 73]. Whereas, the addition of exogenous TGF-*β*3 has in some animal models shown improved scar formation [74]. Further, early human clinical studies showed that exogenous TGF-*β*3 if administered prior to the injury could reduce scarring [75]. However, other studies using a different animal model have shown that TGF-*β*3 had no effect in reducing scar tissue formation [76]. There have been three TGF-*β* receptors identified (T*β*RI, T*β*RII, and T*β*RIII), and variations in the TGF-*β* receptor expressions have been identified in fetal wound healing [8, 77].

Epidermal growth factor (EGF) is known to be involved in wound healing and is thought to be mitogenic for a number of cell types including fibroblasts and keratinocytes. EGF mRNA showed decreased levels with increasing gestational age (scarring) [78]. Surprisingly, the profibrotic platelet-derived growth factor (PDGF) mRNA has also been shown to be elevated in fetal skin compared to adult skin [78] though similar to TGF-*β* it appears to have quicker clearance in fetal wound healing [42]. However, fetal wounds when treated with exogenous growth factors such as PDGF showed a fibrotic response, with increased inflammation, fibroblast recruitment and collagen deposition indicating that fetal wound can respond in an adult manner in response to exogenous PDGF [79]. The fibroblast growth factors (FGF) stimulate proliferation and regulate migration and differentiation in a number of target cells [80]. FGF isoforms are regulated in a complex manner during fetal skin development, and though most do not change expression in scarless healing, both FGF7 and FGF10 were found to be downregulated [60]. The FGF receptor 2 (FGFR2) was down regulated in wound healing, in both scar-forming and scarless healing, but the downregulation was earlier and more sustained in scarless healing [60]. While bFGF (otherwise known as FGF2) and the FGF receptor-1 (flg) expressions were found to be higher in fetal skin than later gestational skin [81].

The role that angiogenesis and in particular VEGF has in scar formation remains unclear. Scarless fetal repair has not only shown reduced angiogenesis in fetal wounds [82], but growth factors associated with angiogenesis show reduced or no expression [25, 70, 79]. Wilgus et al. showed in a murine model that scarless fetal repair heals without either increased VEGF or vascularity [9]. However, other studies have suggested an increase in VEGF mRNA [83]. The variation of the results may be due to the wound model itself, that is, incisional versus excisional, different time and methods used and variations in animal model.

Insulin-like growth factors (IGF) are known profibrotic mitogens known to play a role in wound healing and fetal development. Treating wounds with exogenous IGF-I has been shown to accelerate wound healing through increased collagen synthesis and its mitogenic effect on keratinocytes and fibroblasts [84, 85]. IGF-1 has been implicated in fibrotic conditions including pathological scars possibly due to the increase in collagen synthesis [86, 87]. However, human fetal fibroblasts showed a lower mitogenic response to IGF-I and with a lower level of collagen synthesis compared to adult fibroblasts [88].

## 6. Cell Signalling, Transcription, and Gene Expression

Fetal wound healing and fetal derived cells have indicated that there may be differences in intracellular signalling following the binding of the ligand (growth factor) to its receptor. Martin et al. [70] demonstrated that TGF-*β*1 is rapidly cleared from fetal scarless wounds. While others have shown that the phosphorylation of receptors and some intracellular signalling proteins differ between fetal and adult fibroblasts [68, 88, 89]. The TGF-*β*1 signalling pathway has been shown to be short lived in human fetal fibroblasts after stimulation with exogenous TGF-*β*1 [68], while others found no difference [90, 91]. Variation in results between the studies may be explained through different species (human and mouse) and different intracellular proteins studied (Smad 2 or Smad 3 or Smad 2/3).

Wound healing requires the expression of a number of genes which are regulated by a number of transcription factors such as activator protein 1 (AP1) and the Hox genes. The AP-1 transcription factor is a heterodimeric protein composed of Fos, and Jun and activating transcription factor protein family members. AP-1 induction has been demonstrated in fetal mouse skin, while c-Fos protein was demonstrated to be upregulated in the epidermis after wounding [62, 92]. The increase in AP-1 and c-Fos is linked to Rho, a GTPase, which is linked to the formation of the actin cable in fetal wound closure. Others, have also shown that AP-1 transcription factors were induced after wounding in both scarless and scarring wounds. However, c-fos and c-jun induction was transient in fetal skin while AP-1 expression persisted in scarring tissue [93].

Hox protein activity is essential during embryogenesis, and the Hox genes have been implicated in limb regeneration [94, 95]. A number of Hox genes are expressed in both fetal and adult skin [96, 97], however, fetal wounds show an increase in expression of a number of the Hox genes during fetal scarless repair [98, 99].Though HoxB 13 was downregulated in fetal scarless wounds [99] and in an adult model, Hoxb13 knockout animals showed a more fetal-like healing phenotype [100]. 

Gene expression in fetal fibroblasts shows difference gene expression compared to adult fibroblasts in response to TGF-*β*1 in a number of experimental models [101, 102]. Colwell et al. [102] using genomic microarray demonstrated that fetal wounds have greater increased expression in the fraction of genes immediately after injury. As time after injury lengthened, adult wounds showed the fraction of genes with increased expression increasing. By twenty four hours after injury there were fewer genes with differential expression between the fetus and adult, with the majority having greater expression found in the adult wound [102]. Chen et al. [103] showed that there were fifty three genes (0.93%) differentially expressed between early gestational skin and late gestational skin from rats; 27 genes were upregulated including FGF8, follistatin, and 26 genes were downregulated including beta-catenin in fetal skin when compared to adult skin [103].

## 7. Apoptosis, Proliferation, and Migration

A number of studies suggest that fetal fibroblasts proliferate more rapidly than adult fibroblasts [104]. Though others suggest that there is no difference between fetal epidermal proliferation and adult proliferation [105].

Apoptosis is an important process in wound healing occurring in inflammatory cells, myofibroblasts, and vascular cells, for example. Studies have shown that scarless healing shows no difference in total caspase 3 or any cleavage of caspase 3 compared to scarring healing in a murine model. However, scarless healing showed an increase in cleaved caspase 7 after wounding while scar-forming wounds showed no increase. Further scarless healing showed increased levels of cleaved PARP while scar-forming healing only showed a small amount of cleaved PARP [106]. 

## 8. Problems in Fetal Wound-Healing Research

A number of animal models have been used to study fetal wound healing *in vivo* [54, 67, 107]. In addition a number of *in vitro* studies have used human fetal-derived cells [68, 88, 90]. The use of different species in wound healing studies can make direct comparisons either difficult or impossible with different species demonstrating variations in a number of wound-healing processes. Further complications in comparing fetal wound healing are in the wound itself with some studies using incisional wounds, excisional wounds, or even wounds created by burns. Interestingly the ability of the fetus to heal excisional wounds with perfect regeneration has been shown to be species dependent [54, 108]. Further some fetal excisional wounds undergo contraction (sheep) [108] while others show no contraction in closing excisional wounds (rabbits and monkeys) [107, 108]. 

## 9. Conclusion

The precise mechanism of fetal regeneration remains unclear with a number of differences identified between the fetal and adult wound healing (Table 2). A number of potential antiscarring therapeutics have evolved from understanding fetal regeneration though to date none have completely prevented scar formation. Recent studies have further suggested a role for fetal cells in difficult-to-heal wounds [109] through their promoting effect on adhesion, proliferation, and migration of existing cells.

Further work is required to understand how fetal cells promote regeneration and wound healing and if this can be promoted in adult wound repair. Work will also need to study the role that stem cells play in both adult and fetal wound repair. However, understanding fetal wound healing and regeneration will impact adult repair in the future and may lead to the reduction or even prevention in the formation of scar tissue in a number of organs.

## Figures and Tables

**Table 1:** Differences identified in fetal wound healing compared to adult wound healing.

Growth factor	Role in wound healing	Adult wound healing	Fetal wound healing
EGF	Reepithelisation. Stimulate fibroblasts to secrete collagen	Decreased levels mRNA with increasing gestational age [78]	

VEGF	Angiogenesis		Remains unclear [9, 82]

PDGF	Fibroplasia. Attract fibroblast to wound area.		Elevated levels but quicker clearance from wounds [77]. Exogenous addition causes fibrosis [79]

FGF	Matrix deposition, reepithelisation, angiogenesis, endothelial, keratinocyte, and fibroblast migration		FGF7 and 10 downregulated [60] FGF2 increased expression [81]

TGF-*β*1	Neutrophil infiltration, macrophage infiltration, fibroplasia, matrix deposition, scarring/fibrosis angiogenesis	Increased levels, long intracellular signalling. Causes increase in own gene expression	Low levels with increased clearance [8, 70, 71]. No increase in own gene expression [101]

TGF-*β*2	Neutrophil infiltration, macrophage infiltration, fibroplasia, matrix deposition, scarring/fibrosis angiogenesis		High levels mRNA but not protein [69]

TGF-*β*3	As above but possibly antiscarring	Delayed expression	Increased levels and quicker and prolonged expression [69, 71]

IGF-I	Matrix deposition, scarring, re-epithelisation	Higher proliferation increased collagen synthesis	Lower proliferation and collagen synthesis [88]

**Table 2:** Summary of differences identified in fetal wound healing.

	Fetal wound healing
Inflammation	Reduced immune cells, less activated, lower levels of cytokines, and growth factors due to reduced immune cells [5–9] Decreased expression IL-6 and IL-8 [16, 17] Low levels of COX-2 and PGE2 [25] Appear refractory to exogenous PGE2 [27]

ECM	Higher expression of hyaluronic acid [30, 31] Increased CD44 (hyaluronic acid receptor) [33] Tenascin C earlier deposition [42, 43] Increased expression of some subunits integrins [45] Fibronectin isoforms Reduced decorin [46] Increase fibromodulin [49] Collagen ratio remain unclear but fetal wounds [51, 52] Have reduced cross-linking but increased expression DDR [57] Increased levels of MMPs and urokinase plasminogen activator reduced TIMPs and PAI-1 [58, 59]

Wound closure	Myofibroblasts quick but transitory appearance [67, 68] Close wounds by actin cable [61, 62]

Growth factors	See Table 1

Cell-signalling transcription and gene expression	Difference in phosphorylation in some intracellular signalling pathways [88, 89] Transient increase in AP-1 [93] Hox gene expression differ [98, 99]

Cell behaviour	Increased cleaved caspase 7 Increased cleaved PARP [106]

## Abbreviations

AP-1: Activator protein 1
COX-2: Cyclooxygenase-2
ECM: Extra cellular matrix
EGF: Epidermal growth factor
HA: Hyaluronic acid
IL: Interleukin
MMP: Matrix metalloproteinase
PDGF: Platelet derived growth factor
PGE2: Prostaglandin 2
TGF-*β*: Transforming growth factor-beta.
